# Lifestyle behaviours in patients with established cardiovascular diseases: a European observational study

**DOI:** 10.1186/s12875-019-1051-3

**Published:** 2019-11-26

**Authors:** Diana Fernández, Carlos Brotons, Irene Moral, Mateja Bulc, Mélanie Afonso, Hülya Akan, Susana Pinto, Jasna Vucak, Carlos Manuel da Silva Martins

**Affiliations:** 1 Research Unit. Biomedical Research Institute Sant Pau (IIB Sant Pau), c/ Sardenya 466, 08025 Barcelona, Spain; 2 Teaching Unit of Familiy Medicine ACEBA, Barcelona, Spain; 3 Community Health Centre Ljubljana, Medical Faculty of Ljubljana University, Ljubljana, Slovenia; 4 Département de Médecine Générale (Case 148), Clinique des Universités de Médecine Générale, Bordeaux Cedex, France; 5 Department of Family Medicine, Yeditepe University Faculty of Medicine, İnönü Mahallesi, Kadıköy -, İstanbul, Turkey; 6 Family Medicine, Department of Community Medicine, Information and Decision in Health (MEDCIDS) of the Faculty of Medicine of Porto, Porto, Portugal; 7 Centre for Health Technology and Services Research (CINTESIS), Porto, Portugal; 8 Spec.ord.obiteljske med, Sukosan, Croatia

**Keywords:** General practitioners/family physicians, Cardiovascular diseases, Prevention

## Abstract

**Background:**

Patients who have experienced a cardiovascular clinical event such as a myocardial infarction or stroke qualify for intensive risk factor evaluation and management.

The aim of this study is to explore lifestyle changes as well as the achievement of targets for risk factors in patients with established cardiovascular disease.

**Methods:**

Cross-sectional study conducted in primary care practices. The study was carried out in six European countries (Croatia, France, Portugal, Slovenia, Spain and Turkey). Patients with established cardiovascular disease (coronary heart disease and stroke) attended in primary care were selected and assessed from January to June 2016. Patients were recruited and assessed at the practice by research assistants between 6 months and 3 years after the event. Statistical comparisons were done with the unpaired two-sided Student’s t-test for continuous variables and Chi-square test for categorical variables.

**Results:**

Nine hundred and seventy-three patients (32.4% females) were assessed. About 14% of them were smokers, 32% were physically inactive, and 30% had nutritionally poor eating behaviours. LDL cholesterol target value below 70 mg/dl was achieved in about 23% of patients, and in general, women were less cardio-protected by drugs than men.

**Conclusions:**

Many patients with established cardiovascular disease who attended in general practice still fail to achieve the lifestyle, risk factor, and therapeutic targets set by European guidelines. These results are relevant to general practitioners because these patients have a high risk of subsequent cardiovascular events, including MI, stroke, and death.

## Background

Behaviours such as smoking, a nutritionally poor diet, excessive alcohol consumption, and physical inactivity are the most important modifiable risk factors for the prevention of chronic diseases which currently account for over 70% of the overall global burden of disease, and are expected to rise to 80% by the year 2020 [1].

According to EUROSTAT data, in 2017, nearly 38% of people in the European Union visited their general practitioner once or twice in the last 12 months. A quarter (25%) consulted their general practitioner 3 to 5 times and 14% reported that they saw their general practitioner 6 times or more, while nearly 24% did not go to see a general practitioner [2].

Therefore, primary care is a perfect setting for interventions to reduce behavioural risks factors and recommend preventive interventions to healthy individuals as well as patients with established diseases.

There are certain interventions designed to improve life expectancy and clinical outcomes in people with established cardiovascular disease (CVD) [3]. Despite their substantial potential to reduce the risk of recurrent disease and death, reports on the implementation of prevention guidelines are disappointing. A survey performed by cardiologists in 27 European countries (EUROASPIRE V) demonstrated that a large majority of coronary patients have unhealthy lifestyles in terms of smoking, diet and sedentary behaviour, which adversely impacts major cardiovascular risk factors [4].

Also, results of intervention studies show that further improvements in risk factor management are difficult to achieve. A cluster-randomised, trial performed in GP/FPs in Ireland, after 18 months of a tailored care intervention for practices and patients with coronary heart disease, resulted in a reduction in hospital admissions, but no improvements in cholesterol or blood pressure (BP) levels, or change in mental or physical health status [5]. Another cluster randomized clinical trial was carried out in a regular general practice setting in different health centres in Spain in 1224 patients aged under 86 years with a diagnosis of ischaemic heart disease, stroke or peripheral artery disease [6]. It was observed that a specific programme for prevention of recurrent cardiovascular disease in general practice was not effective. However, it was observed an improvement in factors associated with a healthy lifestyle, and cases of anxiety and depression were reduced.

European guidelines for CVD prevention advocate that patients who have had a cardiovascular clinical event automatically qualify for intensive risk factor evaluation and management. This includes the adoption of a healthier diet, increasing physical activity, and a prompt intervention on all risk factors [7, 8].

## Methods

### Aim

The aim of this study was to describe lifestyle behaviours such as physical activity, healthy diet, smoking cessation, as well as achievement of targets for cardiovascular risk factors and use of pharmacological treatment in patients with established CVD attended in general practice.

### Design and setting

The study consisted of a cross-sectional study in six European countries (Croatia, France, Portugal, Slovenia, Spain and Turkey) conducted by EUROPREV—the European Network for Prevention and Health Promotion in Family Medicine/General Practice (http://europrevdev.woncaeurope.org/), one of the networks of WONCA Europe. Within each country, a national coordinator selected the practices from a list of GP trainers, colleges, or University departments. Patients were selected from GP/FPs practices from January 1st until June 30th, 2016. Males and females (aged > 18 years and < 85 years) were identified with the following first or recurrent clinical diagnosis of myocardial infarction (MI) (ST elevation and non-ST elevation MI), unstable angina, and ischaemic stroke/transient ischemic attack (defined as a neurological deficit that lasts less than 24 h and there is neuroimaging evidence of new ischemic lesion). Patients were included between 6 months and 3 years after the event. Patients with a severe physical disability or impaired cognitive function, patients visited at home, patients institutionalized in nursing homes, and patients who had not been visited in primary care in the last year were excluded from this study.

Research assistants (including research nurses and trainees) selected the patients, reviewed the medical records (to collect data on tobacco and alcohol consumption before the event, lipid levels and drugs taken after the event) and did face to face assessments to collect data on tobacco and alcohol consumption at the moment of the assessment, as well as data on diet and physical activity. Weight and height were measured to all patients using standardized methods. BP was measure with the appropriate cuff, either with a sphygmomanometer or with an automated device. Patients were asked to refrain from smoking or drinking tea/coffee, exercise for at least 30 min before measuring the BP.

Patients were allowed to sit for at least 5 minutes before beginning BP measurement.

### Questionnaire

A structured questionnaire (Additional file 1) was originally developed by the researchers in English and later translated into each of the participating countries’ languages and was used by research assistants to assess all the participants. The questionnaire contained three sections: the first concerned data on socio-demographic and clinical characteristics; the second part examined lifestyle behaviours including physical activity, diet, smoking, and alcohol consumption; the third section gathered information on patients’ CVD risk factors: BP, lipids and glycated haemoglobin (HbA1C) for diabetics, and use of therapeutics that reduce CVD mortality.

Healthy eating behaviours were assessed by the Mediterranean diet scoring system, developed in 1995 with the aim of providing a practical tool to estimate how closely a population follows the guidelines of the Mediterranean diet [9]. The final form published in 2003 [10, 11] contains nine items, according to their position in the Mediterranean diet pyramid (vegetables, fruits, whole grains, wine, fish, legumes/beans, nuts/seeds, olive oil, red or processed meat). A score of zero was assigned when someone reported non appropriate consumption and one was assigned for appropriate consumption. Thus, the scores range from zero to nine, and higher values indicate greater adherence to the Mediterranean diet.

Physical activity was assessed by the short version of the International Physical Activity questionnaires (IPAQ), a questionnaire validated and translated into different languages in Europe [12]. IPAQ assesses physical activity undertaken across a comprehensive set of domains including leisure time, domestic and gardening (yard work), work-related, and transport-related (www.ipaq.ki.se).

### Data entry

A specific secured website was developed with all the items included in the questionnaire, and online data entry was carried out by national research assistants. Principal investigator had accessed to the full data set once information of the questionnaires was entered and saved at local level. Management of the data respected ethical conditions such as the confidentiality and anonymity of participants.

### Statistical analysis

Considering an estimated true proportion of 0.5 fulfilling guideline recommendations, adopting the most conservative option, a margin error of 7%, a confidence level of 95%, and 15% needed resampling to replace non-responders or non-located patients, the required sample size calculated per country was about 231 patients, for a total sample of 1155 patients.

For comparisons of two independent continuous variables, we used unpaired two-sided Student’s t-test. In these cases, data were described using mean and confidence interval. For comparing categorical variables, we used Chi-square test. In these cases, data were described using frequencies (absolutes and relatives).

A *p* value equal to or less than 0.05 was considered to be statistically significant.

All statistical analyses were performed in the coordinating centre using the STATA statistical software (Version 14, StataCorp, Texas, USA).

## Results

Nine hundred and seventy-three patients (32.4% females and 66.6% males) from six European countries were assessed. Table 1 depicts patients’ demographics and clinical characteristics by sex. Females were significantly older than males, and the percentage of women with no or only primary education was also higher than in males. Sixty three per cent of patients had an MI, 35.1% of patients had a stroke/TIA and 1.5% of patients had both. Males had a higher incidence of MI (63.4% vs 49.5%) while females had a higher incidence of stroke (49.2% vs 35.1%).

**Table 1:** Patient demographics and characteristics by sex

	Males (*N* = 658)	Females (*N* = 315)	*p*-value
Age (years), mean (95% CI)^a^	65.56 (64.67–66.45) (*n* = 630)	69.25 (67.89–70.62) (*n* = 304)	< 0.001
Type of Health Centre, n (%)			
Urban	446 (67.78%)	212 (67.30%)	0.881
Rural	212 (32.22%)	103 (32.70%)	
Education, n (%) [*n* = 902]^b^			
No Studies/Primary	327 (53.78%)	196 (66.67%)	< 0.001
Secondary	193 (31.74%)	80 (27.21%)	
Tertiary	88 (14.47%)	18 (6.12%)	
Employment,% (95% CI) [*n* = 902]^b^			
Employed	207 (34.05%)	60 (20.41%)	< 0.001
Student	3 (0.49%)	1 (0.34%)	
Housewife/husband or equivalent	4 (0.66%)	23 (7.82%)	
Retired	363 (59.70%)	195 (66.33%)	
Unemployed	31 (5.10%)	15 (5.10%)	

^a^There were some missing data for the Age variable

^b^France without data

Table 2 details the prevalence of lifestyle behaviours and cardiovascular risk factors by sex. Males had significantly higher percentages of unhealthy behaviours compared to females. According to the Mediterranean Diet Score, males had worse eating behaviours than females (33.1% vs 22.2%) after the event. On the contrary, according to the IPAQ questionnaire, females were less physically active than males (37.8% vs 28.9%) after the event. Achievement of LDL-cholesterol (LDL-c) target (< 70 mg/dl) was significantly higher in males than in females (25.7% vs 17.1%).

**Table 2:** Lifestyle behaviours and risk factors after the event by sex

		Males (*N* = 658)	Females (*N* = 315)	*p*-value
Lifestyle behaviours				
Smokers, % (95% CI)	Before event	262 (39.82%)	73 (23.17%)	< 0.001
	After event	99 (15.05%)	38 (12.06%)	0.211
Risky drinkers, % (95% CI)	Before event	111 (16.87%)	9 (2.86%)	< 0.001
	After event	44 (6.69%)	4 (1.27%)	< 0.001
Lack of physical activity (IPAQ inactive)		190 (28.88%)	119 (37.78%)	< 0.001
Patients that have been advised to increase physical activity		548 (83.28%)	255 (80.95%)	0.370
Patients that have increased their physical activity?		288 (43.77%)	124 (39.37%)	0.193
Nutritionally poor eating behaviours (Mediterranean diet score 0–3 points)		218 (33.13%)	70 (22.22%)	0.007
Patients that have been advised to change their diet		566 (86.02%)	261 (82.86%)	0.196
Patients that have improved their diet (after event)		458 (69.6%)	208 (66.03%)	0.262
Cardiovascular risk factors				
Overweight/Obesity (BMI > 25)		499 (78.09%) (*n* = 639)	231 (75.99%) (n = 304)	0.505
Diagnosis of hypertension		470 (71.43%)	235 (74.60%)	0.300
Diagnosis of diabetes		211 (32.07%)	80 (25.40%)	0.033
Diagnosis of dyslipidaemia		483 (73.40%)	211 (66.98%)	0.038
Diagnosis of CHD		417 (63.37%)	156 (49.52%)	< 0.001
Diagnosis of Stroke		231 (35.11%)	155 (49.21%)	
Diagnosis of CHD and Stroke		10 (1.52%)	4 (1.27%)	
Control^a^ of blood pressure (< 140/90 mmHg)		486 (73.86%)	239 (75.87%)	0.500
Control^a^ of LDL cholesterol (< 70 mg/dl)		169 (25.68%)	54 (17.14%)	0.003

^a^Missing values were considered as no control. *CHD* Coronary heart disease. *IPAQ* International Physical Activity questionnaire. *BMI* Body Mass Index

Regarding pharmacotherapy, Table 3 shows that males were more protected by therapeutic compounds than females, except for Angiotensin-converting-enzyme inhibitors (ACEi) and Angiotensin II receptor blockers (ARBs). Changes in lifestyle behaviours after the event include a reduction in smoking and in alcohol consumption in both sexes (Table 2). According to patients’ perception, a large proportion of patients were advised by GPs to increase their physical activity and to change their diet after the event; about two-thirds of the patients acknowledge having changed their diet while less than 50% acknowledge having increased their physical activity after the event.

**Table 3:** Pharmacotherapy after the event

	Men (*n* = 608)	Women (*n* = 294)	*p*-value
Betablockers, n (%)	368 (60.53%)	155 (52.72%)	0.026
ACEi/ARBs, n (%)	450 (74.01%)	216 (73.47%)	0.862
Statins, n (%)	544 (89.47%)	241 (81.97%)	0.002
Antiplatelet, n (%)	530 (87.17%)	236 (80.27%)	0.007
Anticoagulants, n (%)	58 (9.54%)	53 (18.03%)	< 0.001

*ACEi* Angiotensin-converting-enzyme inhibitor, *ARBs* Angiotensin II receptor blockers

## Discussion

### Main results

Our study reveals that many patients with established cardiovascular disease attended in primary care still fail to achieve the lifestyle, risk factor, and therapeutic targets set by the European guidelines [7]. About one-third of men and one-half of women who smoked before the event were still smokers at follow-up About two-thirds of the patients in the survey reported changing their diet since the event, but only half reported increasing physical activity.

The majority of the patients assessed were overweight or obese, contributing to the high prevalence of diabetes recorded in this study. About three-fourths of patients had reached the target for blood pressure of < 140/90 mmHg, and one-fourth of men and less than one-fifth of women reached the target for LDL-c of < 70 mg/dl (< 1.8 mmol/l) recommended in patients with established cardiovascular disease. The majority of the patients are taking at least one of the cardio-protective drugs (beta-blockers, ACE inhibitors/ARBs, statins, anti-platelets), although women were less cardio-protected.

### Results from other studies

The EUROPASPIRE V survey [4] showed that the target of blood pressure and LDL-c were not achieved by 42 and 71% of the coronary patients, respectively. In our survey we found that the target blood pressure was not achieved in only 25% of patients, but the target for LDL-c was not achieved in 77% of patients. General practitioners are probably more familiar with treatment and control of hypertension as a risk factor than with treatment of hypercholesterolemia. The inadequate lipid control observed may include starting with a low dose and not up-titrating or poor patient adherence; the latter is associated with higher rates of cardiovascular events, all-cause mortality, and health care costs. A systematic review and meta-analysis of prospective epidemiological studies demonstrated that about 9% of all CVD events in Europe could be attributed to poor compliance to cardiovascular drugs and that optimal levels of compliance confer a statistically significant inverse association with subsequent adverse outcomes [13]. Therefore, measures to improve compliance in primary care should help maximize the potential of effective cardiovascular drugs.

It is also important that patients with established cardiovascular disease receive professional advice from GP/FP teams on healthy eating and safe increases in physical activity to reduce the prevalence of obesity and overall risk.

An observational study in patients that suffered an acute coronary syndrome showed that adherence to diet, exercise, and smoking cessation was associated with an important lower risk of recurrent cardiovascular events [14], compare to non-adherence.

### Strengths and limitations

The study has some limitations that should be mentioned. European primary professionals that participated in this study were motivated to address lifestyle risk factors compared to other groups and also may not have selected a representative sample of patients for each country.

Also, we included only patients that have been visited in the consultation in the last year. Patients that are not visited regularly by their GPs may have even worse results in terms of modifying unhealthy behaviours or treated with appropriate drugs.

However, this multinational study has many strengths including that it followed one protocol and used standardised methods, was carried out in a GP/FP setting, and included coronary heart disease patients and stroke patients.

### Conclusions

A substantial proportion of patients with established cardiovascular disease visited in primary care have not modified their lifestyle, and do not reach target levels of blood pressure and lipids recommended by guidelines. These results are relevant to general practitioners because these patients have a high risk of subsequent cardiovascular events. Therapeutic lifestyle changes such as increased physical activity, dietary modification/weight loss, and smoking cessation are of proven benefit and are likely to improve outcomes beginning within a matter of months.

A result that also should be of interest to GPs is that there are adjunctive drug therapies of proven benefit in these patients, such as aspirin, statins, beta blockers and ACEi or ARBs that should be used in both male and females. The LDL-c target of < 70 mg/dl is achieved by about 23% of patients, and in general, females are less cardio-protected by drugs than males. Further research is needed to investigate the reasons behind these results so that specific strategies may be devised to address them.

## Supplementary information

**Additional file 1.** Questionnaire in English version used to collect study data.

## Data Availability

The datasets used and/or analysed during the current study are available from the corresponding author on reasonable request.

## Abbreviations

BP: Blood pressure
CVD: Cardiovascular disease
HbA1C: Glycated haemoglobin
IPAQ: International Physical Activity questionnaires
LDL-c: LDL colesterol
MI: Myocardial infarction
