# Supplementary material for: Lifestyle behaviours in patients with established cardiovascular diseases: a European observational study
Source: BMC Fam Pract. 2019 Nov 26;20:162. doi: 10.1186/s12875-019-1051-3 (PMC6878626; doi:10.1186/s12875-019-1051-3)
Supplement: Supplementary file 1 — Additional file 1. Questionnaire in English version used to collect study data. [file 12875_2019_1051_MOESM1_ESM.pdf]

Questionnaire
Lifestyle habits in patients with established cardiovascular diseases (CVD).

"EUROPREV III" Study

Date of the visit: ___/___/___
Country____________
Primary care center: __________________
Rural_(<10,000 resident population) ___ Urban____
Patient Nº.: ________
Gender: M___ F___
Education: No Studies/Primary__ Secondary__ Tertiary__
Employment: Employed__ Student__ Housewife __ Pensioner__ Unemployed__
Date of Birth: ___/___/___ (day/month/year)

Medical History:
Hypertension: Yes___ No___
Diabetes: Yes___ No___
Diabetes mellitus type I: ___ Yes___ No___
Diabetes mellitus type II: ___ Yes___ No___
Dyslipidemia: Yes___ No: ___

Cardiovascular disease: (One of these events has to be diagnosed between 6 months and 3 year)
Unstable angina: Yes ___ No___ Date: ___/___/___
Myocardial Infarction: Yes ___ No___ Date: ___/___/___
Ischemic Stroke (included TIA): Yes___ No___ Date: ___/__/___

LYFE STYLE HABITS

TOBACCO:

1. Were you a smoker when you had the CV event? Yes___ No___
   1. a How many cigarettes did you smoke per day? ___
2. Do you currently smoke? Yes___ No___
   2. a How many cigarettes do you smoke per day? ___
   2. b How many cigars do you smoke per day? ______
   2. c Have you tried to quit smoking? Yes___ No___
3. Have you been advised to attend a smoking cessation clinic? Yes____No_____
4. Have you been advised to use pharmacologic support? Yes______No______
   If Yes, what kind of pharmacologic support:
   4. a Nicotine- replacement therapy: Yes_____No_______
   4. b Varenicline: Yes______No_______
   4. c Bupropion: Yes_______No_______

ALCOHOL CONSUMPTION: (Consumption per day)
(one unit -10gr of alcohol- is: 250ml of beer, 100ml of wine or champagne, 50ml of cherry, and 25ml or rom, gin, whisky or vodka)

1. Did you drink alcohol before CV event? Yes___ No___
   If Yes
   1. a Beer_____ Units/week
   1.b Wine_____ Units/week
   1.c Others__________________ Units/week.

2. Do you currently drink alcohol? Yes___ No___
   If Yes
   2.a Beer_____ Units/week
   2.b Wine_____ Units/week
   2.c Others__________________ Units/week

PHYSICAL ACTIVITY

1. Have you been advised from a health professional to increase your physical activity?
   Yes_______No_______

2. Have you increased your physical activity since your cardiovascular event?
   Yes_______No_______

IPAQ – Short form.
(Time patients spent being physically active after CV event)

1. During the last 7 days, on how many days did you do **vigorous physical activities** like heavy lifting, digging, aerobics, or fast bicycling? (At least 10min at a time)

   _____Days per week. (If none put 0)

   1. a How much time in total did you usually spend on one of those days doing vigorous physical activities?

   Hours______ Minutes_____

2. During the last 7 days, how many days did you do **moderate physical activities** like carrying light loads, bicycling at a regular pace, or doubles tennis? Do not include walking. (At least 10 min at a time)

   _____Days per week (If none put 0)

   2.a How much time in total did you usually spend on one of those days doing moderate physical activities?

   _____Hours Minutes_____

3. During the last 7 days, on how many days did you **walk** for at least 10 minutes at a time? This includes walking at work and at home, walking to travel from place to place and any other walking that you did solely for recreation, sport, exercise or leisure.

   _____Days per week

   3.a How much time in total did you usually spend walking on one of those days?

   _____Hours Minutes_____

4. The last question is about the time you spent **sitting** on weekdays while at work, at home, while doing course work and during leisure time. This includes time spent sitting at a desk, visiting friends, reading traveling on a bus or sitting or lying down to watch television.

   During the last 7 days, how much time in total did you usually spend sitting on a week day?

   _____Hours Minutes_____

EATING HABITS

1. Have you been advised from a health professional to change your diet?
   Yes_______No_______

2. Have you improved your diet since your cardiovascular event?
   Yes_______No_______

MEDITERRANEAN DIET SCORE

		Yes (1)	No (0)
Vegetables	Two or more cups a day		
Fruit	Two or more pieces a day		
Whole grains	Two or more whole grains a day		
Wine	½ to 1 drink for women, 1-2 for men a day		
Fish	Two or more times a week		
Legumes/Beans	Two or more servings a week		
Nuts/Seed	A handful of nuts most days		
Fat	Olive oil and few other fats		
Red or Processed Meat	Two servings or fewer a week		

CARDIOVASCULAR RISK FACTORS.

Biometric Measures: (measure BP twice in upper right arm in a sitting position, record mean value)

SBP____ DBP____
Height: ____cm
Weight: ____kg or ____pounds
Abdominal Circumference: ___cm

Blood test date: ___/___/___ (last available in the medical history, > 3 months after the event and performed in the last year.))

Total Cholesterol (mmol/l) _____mg/dl_____		Triglycerides mmol/l____mg/dl____	
LDL Cholesterol (mmol/l)_____mg/dl______		Glucose (mmol/l)_____mg/dl______	
HDL Cholesterol (mmol/l)_____mg/dl______		HbA1c (%)	

List of medication you are currently taking.

	Yes (1)	No (2)
Aspirin (mg/day)		
If YES, record dosage		
Dipyridamole (mg/day)		
Clopidogrel (mg/day)		
Other antiplatelet		
If YES, record name and dosage		
Oral anticoagulants (mg/day)		
Heparin (mg/day)		
Beta blockers (mg/day)		
Statins		
If YES, record name and dosage		
Other lipid lowering drugs (mg/day)		
ACE inhibitors(mg/day)		
If YES, record name and dosage		
AT II receptor blockers(mg/day)		
If YES, record name and dosage		
Diuretics (mg/day)		
Other antihypertensive (mg/day)		
